# Analyse de la prise en charge du nouveau-né dans le cadre de la stratégie nationale de subvention des accouchements et des soins obstétricaux et néonatals d'urgence au Centre Hospitalier Universitaire Pédiatrique Charles de Gaulle, Ouagadougou (Burkina Faso)

**DOI:** 10.11604/pamj.2015.20.176.5891

**Published:** 2015-02-25

**Authors:** Solange Odile Yugbaré Ouédraogo, Nestor Yougbaré, Fla Kouéta, Dao, Moussa Ouédraogo, Claudine Lougué, Kam Ludovic, Ramata Ouédraogo Traoré, Diarra Yé

**Affiliations:** 1 Unité de Formation et de Recherche en Sciences de la Santé (UFR/SDS), Université de Ouagadougou, Ouagadougou, Burkina Faso; 2 Centre Hospitalier Universitaire Pédiatrique Charles de Gaulle, Burkina Faso; 3 Service de Pédiatrie du CHU-YO, Ouagadougou, Burkina Faso

**Keywords:** Subvention, qualité des soins, nouveau né, morbidité, décès, subsidy, quality of care, newborn, morbidity, deaths

## Abstract

**Introduction:**

Il s'agit d'analyser la prise en charge du nouveau-né dans le cadre de la stratégie na-tionale de subvention des accouchements et des soins obstétricaux et néonatals d'urgence mis en place par le gouvernement du Burkina Faso en 2006.

**Méthodes:**

Nous avons menée une étude à visée descriptive et analytique comportant un volet ré-trospectif du 01 janvier 2006 au 31 décembre 2010 portant sur les paramètres épidémiologiques, cliniques des nouveau-nés hospitalisés et un volet prospectif du 3 octobre 2011 au 29 février 2012 par une entrevue des accompagnateurs des nouveau-nés et des prestataires des services de santé.

**Résultats:**

Les hospitalisations ont augmenté de 43,65% entre 2006 à 2010 Le taux de mortalité néo-natale hospitalière qui était de 11,04% a connu une réduction moyenne annuelle de 3,95%. L'entrevue a porté sur 110 accompagnateurs et 76 prestataires. La majorité des prestataires (97,44%) et des ac-compagnateurs (88,18%) étaient informés de la stratégie mais n'avait pas une connaissance exacte de sa définition. Les prestataires (94,74%) ont signalé des ruptures de médicaments, consommables médicaux et des pannes d’ appareils de laboratoire et d'imagerie. Parmi les accompagnateurs (89%) disaient être satisfaits des services offerts et (72,89%) trouvaient les coûts abordables mais évoquaient les difficultés du transport. Conclusion: La subvention a amélioré la prise en charge du nou-veau-né mais son optimisation nécessiterait une meilleur information et implication de tous les acteurs.

**Conclusion:**

La subvention a amélioré la prise en charge du nouveau-né mais son optimisation nécessiterait une meilleur information et implication de tous les acteurs.

## Introduction

La mortalité néonatale demeure un problème majeur de santé publique dans les pays en développement. Selon l'Organisation Mondiale de la Santé (OMS), parmi les 11 millions de décès infanto-juvéniles annuels, on compte 4 millions de nouveau-nés et 75% meurent au cours de la première semaine [1] dont 98% en Afrique Sub-saharienne et en l'Asie du Sud-Est. Le quatrième objectif du millénaire pour le développement (OMD4) qui vise à réduire de deux tiers la mortalité des enfants de moins de 5 ans d'ici 2015 ne pourra être atteint sans la réduction d'au moins de moitié la mortalité néonatale [2–4]. Face à cette situation, le gouvernement burkinabè a décidé de subventionner à hauteur de 80%, les soins obstétricaux et néonatals d'urgence (SONU) à partir de l'année 2006 dans toutes les structures sanitaires [5]. Après 5 ans de mise en œuvre, il nous a paru opportun de faire une analyse afin d'optimiser la stratégie au sein de notre structure et de contribuer à une accélération de la réduction de la mortalité néonatale.

## Méthodes

Il s'agit d'une étude rétrospective de type descriptif d'une série de cas du 1er janvier 2006 au 31 décembre 2010 (60 mois), portant sur les paramètres épidémiologiques et cliniques des nouveau-nés, de 0 à 28 jours, hospitalisés au centre hospitalier universi-taire pédiatrique Charles de Gaulle (CHUP-CDG). Les variables suivantes maternelles, paternelles (état civil) et néonatales (diagnostic, évolution) ont été spécifiquement étudiées. Nous avons aussi mené une étude à visée descriptive et analytique transver-sal portant sur l'interview des accompagnateurs des nouveau-nés hospitalisés et des prestataires de service de santé du 03 octobre 2011 au 29 février 2012. Les concepts d'analyse suivant ont été évalués: niveau de connaissance de la subvention, niveau de satisfaction par rapport aux services offerts, coûts. Par ailleurs les indicateurs suivant ont été mesurés: Taux d'occupation des lits, taux de couverture de la subvention, taux de réalisation des examens, taux de prescription des médicaments, taux de mortalité néonatale hospitalière. Le test de Chi-carré et de Fischer ont été réalisé pour comparer les variables catégorielles avec un seuil de significativité à 5%.

## Résultats

**Description des échantillons:** les médecins et stagiaires internés de médecine représentaient la moitié des prestataires inter-rogés comme le montre le Tableau 1. Nous avons interrogés 110 accompagnateurs comportant 75,45% de mères et 14,55% de pères. Les nouveau-nés de mères femmes au foyer (68,15%) et de pères travaillant dans le secteur informel (27,08%) étaient majoritaires. Une mère sur 3 (37,38%) avaient un niveau d'instruction du secondaire et la moitié d'entre elles (57,27%) étaient mariées. Les nouveau nés étaient issus de familles de bas niveau socio-économiques dans 37,27% des cas avec une taille moyenne du ménage de 5,73 personnes et une taille moyenne de la fratrie de 1,67 enfants.

**Tableau 1:** Profil des prestataires interviewés au Centre Hospitalier Universitaire Pédiatrique Charles de Gaulle

Qualification	Effectifs	Fréquence (%)
Médecins et stagiaires internés	38	50,00
Infirmiers	18	23,68
Surveillants d'unité	4	5,26
Pharmaciens et stagiaires internés	4	5,26
Techniciens de laboratoire	4	5,26
Manipulateurs de Radiologie	2	2,63
Agents du service d'information médicale	2	2,63
Préparateurs d'Etat en pharmacie	2	2,63
Technologistes biomédicaux	2	2,63
TOTAL	76	100

**Figure 1:**
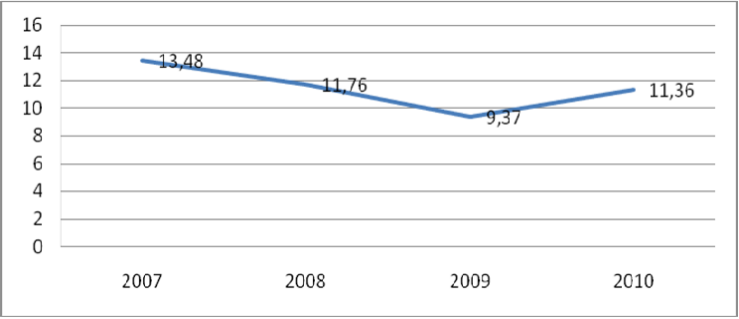
Évolution annuelle de la mortalité hospitalière néonatale de 2007 à 2010

**Morbidité et mortalité néonatale hospitalière:** l'infection néonatale était le diagnostic principal avec 76,02% de cas suivie de la souffrance néonatale 19,79% et de l'hypotrophie 7,78%. La mortalité néonatale hospitalière est passée de 13,48% en 2007 à 11,36% en 2010 (Figure 1). Le taux moyen de réduction annuelle de la mortalité hospitalière était de 3,95%. Le nombre moyen d'hospitalisations par an dans notre série est de 566,8 avec une augmentation annuelle progressive. Le moyen de transport le plus utilisé était la mobylette personnelle dans 38,82% des cas,

**Niveau de connaissance de la subvention des SONU des interwievés (prestataires et accompagnateurs):** nous avons interrogés 78 prestataires, 97,44% d'entre eux étaient informés de l'existence de la subvention. Les médias ont été la principale source d'information (42%) suivi des cadres de rencontre (22%) et de l'hôpital (22%). Le délai moyen de l'information était de 3 ans avec des extrêmes de 4 mois à 7 ans. Le niveau de connaissance était bon pour les rubriques des SO-NU, acceptable pour les buts et objectifs et faible pour les bénéficiaires. Une formation géné-rale sur la prise en charge des nouveau-né concernait un agent sur 4 (20,51%) avec une fréquence variable de une par semestre à une tous les 2 ans. Quant à la formation sur la sub-vention elle n'a été effective que chez 10 prestataires avec des fréquences variable de une fois à une fois par an. L'observation a été faite chez 58 prestataires qui étaient tous en tenue correcte de travail avec un comportement à l'accueil jugé satisfaisant; 97,3% d'entre eux ont salué les accompagnateurs, ces salutations ont été considérées comme respectueuses dans 85,71% des cas. Les examens complémentaires et les traitements ont été expliqué aux accompagnateurs par 80,56% des prestataires. Nous avons interrogé 110 accompagnateurs; 88,18% avaient reçu l'information sur la sub-vention; les sources d'information étaient l'hôpital lors de l'accouchement (53,33%), les parents et amis (33,33%) et les cadres de concertation (13,33%). Le délai moyen de connaissance de l'information était de 11 mois avec des extrêmes d'une semaine à 2 ans. Seuls 3 accompagnateurs avaient une connaissance des bénéficiaires, des rubriques de la subvention et des buts et objectifs.

**Figure 2:**
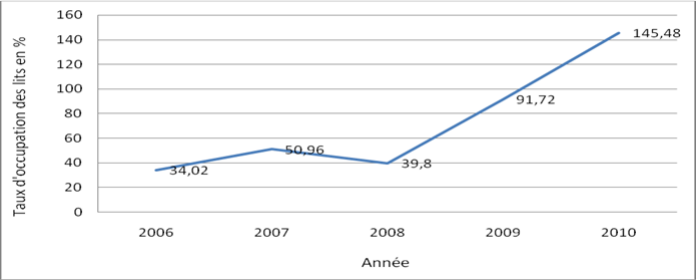
Évolution annuelle du taux d'occupation des lits par les nouveau-nés à l'Unité de Néonatologie

**Capacité fonctionnelle des services:** le taux d'occupation des lits de 2006 à 2010 était de 72,4% avec une croissance constante (Figure 2). La subvention des SONU a couvert plus de 100% des bénéficiaires à partir de 2008 avec un taux moyen de couverture annuelle de 128,04%. Le fonctionnement des services était entravé par la rupture des médicaments, consommables médicaux et les pannes des appareils de laboratoire et de l'imagerie qui ont été signalées par 94,74% des prestataires. La durée moyenne de la rupture était de 34,54 jours avec des extrêmes d'une semaine à une année.

**Figure 3:**
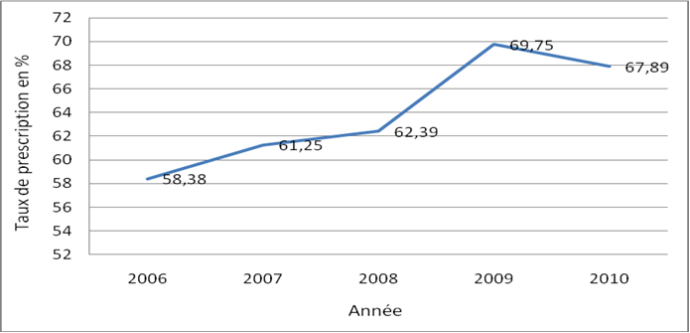
Évolution annuelle du taux de prescription des médicaments dans le cadre des SONU

**Qualité des soins:** le délai moyen de prise en charge était de 99,71 minutes avec des extrêmes de 0 à 850 mi-nutes. Le taux de réalisation globale des examens est resté stable au fil des années entre 34,73% et 48,72%. Le taux moyen de prescription des médicaments était de 64,98%. Il y'a une augmentation du taux de prescription des médicaments de 2006 à 2010 (Figure 3). Sur les 132 nouveau-nés qui ont reçu une prescription de transfusion, 127 (96,21%) ont été transfusé. Les visites médicales des malades étaient régulières durant toute la semaine avec une mise à jour des dossiers et un réajustement du traitement selon les prestataires. Cependant sur 1018 observations, 580 soit 56,97% avaient un traitement irrégulier dû à des difficultés de la reprise de la voie veineuse périphérique(41,38%), aux ordonnances non honorées (41,03%) ou à la non prescription des médicaments (12,24%). Les autres causes retrouvées étaient les ruptures des médicaments, consommables médicaux et la surcharge de travail. Les demandes de sortie étaient significati-vement élevées lorsque le traitement était irrégulier (11,9%) avec une différence qui était si-gnificative p =0,0096.

**Appréciation des coûts:** le transport et les médicaments ont été significativement trop chers pour les accompagnateurs de nouveau-nés issus de famille à niveau socio-économique faible (Tableau 2).

**Tableau 2:** Relation entre le niveau socio-économique des accompagnateurs et l'appréciation des coûts de la prise en charge des nouveau-nés

Item	Niveau socio-économique bas	p
**Transport**		
Trop cher	68,75%	0,0001
Cher	0%	
Abordable	13,33%	
**Hospitalisation**		
Trop cher	0%	0,8
Cher	37,50%	
Abordable	14,29%	
**Médicaments**		
Trop cher	100%	0,004
Cher	0%	
Abordable	13,79%	
**Examens complémentaires**		
Trop cher	0%	0,4
Cher	9,68%	
Abordable	22,22%	

**Niveau de satisfaction:** concernant les différentes composantes du séjour hospitalier, 84,58% des accompagnateurs étaient satisfaits ou très satisfaits. Néanmoins 15,42% étaient insatisfaits ou peu satisfaits des prestataires (Tableau 3). Les plus grandes satisfactions étaient en faveur: des médecins pour leur accueil, l’écoute, la visite et les conseils; les soins surtout administrés par les infirmiers, l’équipement des ur-gences; la propreté des chambres et de la cour; la guérison ou l'amélioration de la santé du bébé et tout cela grâce à la subvention.

**Tableau 3:** Synthèse du niveau de satisfaction des accompagnateurs

Items	Insatisfait (%)	Peu satisfait(%)	Satisfait (%)	Très Satisfait(%)
Prestataires	5,82	14,36	30	49,82
Soins	9,83	7,94	30,06	52,17
Organisation	5,3	4,36	34,58	55,76
Hôtellerie	5,84	4,21	29,44	60,51
Coûts	16,83	2,64	31,35	49,17
Moyenne	8,72	6,70	31,09	53,49

**Contribution de la subvention dans la prise en charge globale du nouveau-né:** la subvention a été d'un grand apport pour plus de la moitié des patients (64%). Selon les accompagnateurs 96,36% conseilleront à leurs connaissances la fréquentation du CHUP-CDG et 90,91% accepteraient revenir pour des services.

**Problèmes énumérés par les interviewés:** la plus grande difficulté rencontrée par les prestataires (63,33%) demeure la rupture des mé-dicaments et des consommables médicaux. Le manque d'hygiène des sanitaires (93,26%) et le défaut de communication du personnel (67,42%) ont été les principales insuffisances men-tionnées par les accompagnateurs.

## Discussion

### Morbidité et mortalité néonatales

Le nombre moyen d'hospitalisations par an dans notre série est de 566,8 avec une augmentation annuelle progressive des hospitalisations. Il est supérieur à celui de Kouéta et coll au Burkina Faso en 2007 [6] qui trouvaient une moyenne de 245 nouveau-nés hospitalisés par an. Ce niveau élevé des hospitalisations pourrait s'expliquer par l'amélioration des condi-tions de l'accessibilité aux services grâce à la subvention car la pauvreté était le principal frein à la fréquentation des services de santé [5] Le moyen de transport le plus utilisé était la mobylette personnelle. Agossou et coll au Bénin [7], Azoumah et coll au Togo en 2010 [8] ont noté aussi que la plupart des transports du nou-veau-né n’étaient pas médicalisés. Ces moyens de transport non adaptés exposeraient le nou-veau-né aux infections, à la détresse respiratoire et à l'hypothermie. En effet Kouéta [9] au Burkina en 2011 trouvait que le risque de décès était 2,8 fois plus élevé lorsque les moyens de transport suivant (moto, taxi, véhicule personnel) remplaçaient l'ambulance d'où la nécessité de mettre en place un système de transport médicalisé pour garantir une meilleure survie du nouveau-né. L'infection néonatale et la souffrance cérébrale néonatale ont été les principales causes de morbidité dans notre série. Le même constat a été faits par plusieurs auteurs [6, 8, 9]. Le taux de mortalité néonatale était de 11,04%. Nos résultats sont inférieurs à la plupart des séries: Azoumah et coll à Dapaong au Togo en 2007 trouvait 24,9% [10]; Madiabala-Babela à Brazzaville en 2009 trouvait 37,4% [11]. Cependant, nos données sont difficilement comparables à celles de ces auteurs du fait de la différence des contextes de travail et des périodes d’étude. Néanmoins, la faiblesse du taux de mortalité dans notre étude s'expliquerait par l'existence d'autres centres de références de prise en charge du nouveau-né prématurés, mais aussi par l'amélioration de la qualité des soins grâce à la mise en œuvre effective de la stratégie des SONU. En effet, avant cette mise en œuvre de la subvention, Kouéta et coll trouvait un taux de mortalité de 15,3% de 2002 à 2006 [6] soit une réduction de la mortalité de 4,26% par rapport à notre série. Cependant, le taux de réduction de cette mortalité nous paraît faible entre 2007 et 2010. L'atteinte de l'OMD4 passe donc par une accélération de la mise en œuvre des différentes stratégies d'amélioration de la santé des enfants. Les causes de décès dans notre étude ont été l'infection néonatale (73,91%) suivis des malformations (14,49%) et des détresses respiratoires (7,25%), le même constat a été fait au Congo par Ma-diabala et coll qui notait (46,8%) de décès par infection néonatale [11]. Cependant Azoumah au Togo [10] et Sylla au Mali [12] notaient la prématurité comme la première cause de décès avec respectivement (32,62%) et (42,9%).

### Niveau de connaissance de la subvention des SONU des interviewés

Dans notre série la quasi-totalité des prestataires (97,44%) étaient informés de l'existence de la subvention. Nos résultats sont similaires à ceux de Zombré au Burkina Faso en 2009 ou tous les prestataires étaient informés [13]. La principale source d'information était les médias dans notre série alors que pour Zombré la voie administrative se révélait être la meilleure. Cependant, aucun prestataire n'avait une connaissance exacte du contenu de la subvention d'où la nécessité d'une meilleure diffusion des informations au sein de la structure. L'information sur la subvention était connue par 88,18% des accompagnateurs. Pour Sombié et coll, 47,6% des accompagnateurs avait l'information sur la subvention en 2007 [14]. Cette augmentation témoigne des efforts de sensibilisation entrepris depuis la mise en œuvre de la Stratégie. La principale source d'information reste pour la plupart des études les services de santé [13, 14], d'où la nécessité d'une meilleure implication des médias pour la sensibilisation de la communauté.

### Capacité fonctionnelle des services

Le taux d'occupation des lits en néonatologie était supérieur au taux global d'occupation de l'ensemble des hospitalisations au CHUP-CDG en 2010 et reste le plus élevé au niveau national [15]. Une meilleure organisation avec une augmentation de la capacité d'accueil et des ressources humaines s'avère urgente. La rupture des stocks de médicaments, de consommables médicaux, et les pannes d'appareils de laboratoire et d'imagerie étaient très fréquentes. Les principales causes étaient la complexité de la procédure administrative des marchés, un défaut de maintenance préventive et curative des appareils. Il en résulte des difficultés pour l'application adéquate de la subven-tion affectant la qualité des soins car les patients sont obligés de faire recours aux officines et laboratoires de ville augmentant ainsi le délai et le coût de la prise en charge. Ces ruptures intempestives sont responsables du faible taux de réalisation des examens complémentaires. L'amélioration de la qualité des prestations passe par la révision des méthodes de gestion des stocks, des procédure de commande des médicaments, consommables médicaux et la mainte-nance adéquate des appareils. Aussi, une formation des prestataires sur la subvention est nécessaire pour une appropriation des protocoles et une rationalisation de la prescription des médicaments et consommables.

### Qualité des services offerts

La majorité des utilisateurs étaient satisfaits ou très satisfaits de l'accueil (80%) comme dans l’étude de Zombré (96,6%) [13]. Les relations interpersonnelles et entre soignants et soignés étaient en générale satisfaisantes. Cependant, l'insuffisance de communication et d’écoute de la part des prestataires a été notée par 67,42% des accompagnateurs. Cissé dans une étude sur la gratuité de la césarienne au Mali a trouvé que le comportement des agents de santé était désagréable dans les formations sanitaires [16]. Les mauvais comportements réduiraient la fréquentation des services de santé avec un impact négatif sur la stratégie de réduction de la mortalité maternelle et néonatale.

### Qualité des soins

Les utilisateurs étaient satisfaits ou très satisfaits du délai de prise en charge (87,48%) comme dans l’étude de Zombré (91,6%) [13]. Ces résultats traduiraient l'effort des prestataires pour satisfaire leurs clients. Toutefois, on pourrait réduire le délai de prise en charge par le renforcement des compétences des ressources humaines. Quant à la continuité des services, les visites sont assurées sept jours sur sept mais plus de la moitié des patients ont un traitement irrégulier. Les principales raisons sont les difficultés de la prise de la voie veineuse périphérique du nouveau-né et les ordonnances non honorées. Ces situations interpellent à reconsidérer la place de la voie veineuse périphérique chez le nouveau-né qui est peu durable par rapport au cathétérisme veineux ombilical et à une meilleur gestion des stocks de médicaments et consommables. Malgré ces obstacles, 82,23% des accompagnateurs étaient satisfaits des soins mais nos résultats restent inférieurs à ceux de Zombré et Oyo au Nigéria qui trouvaient respectivement 99,8% et 97,2% [13, 17]. Ces chiffres doivent être pris en compte pour l'amélioration de la qualité des services.

### Niveau globale de satisfaction des accompagnateurs

Dans notre étude, 90% des accompagnateurs étaient satisfaits ou très satisfaits de leur séjour hospitalier. Cela explique l'importance de la sollicitation du service et appel à des prospections pour l'augmentation de la capacité d'accueil. En effet, plus de 90% des accompagnateurs ont promis de revenir pour des soins à l'hôpital et de donner des conseils à leurs connaissances pour les inciter à fréquenter la structure.

## Conclusion

La stratégie nationale de subvention des soins obstétricaux et néonatals d'urgence mise en œuvre au CHUP-CDG depuis 2006 est assez bien connue des prestataires mais peu connue des accompagnateurs. Le renforcement des ressources humaines et matérielles permettraient d'optimiser la mise en œuvre de la stratégie SONU.
